# A Systematic Review of Location Aware Schemes in the Internet of Things

**DOI:** 10.3390/s21093228

**Published:** 2021-05-06

**Authors:** Muneeb A. Khan, Abdul Saboor, Hyun-chul Kim, Heemin Park

**Affiliations:** 1 Department of Software, Sangmyung University, Cheonan 31066, Korea; muneebkhan046@gmail.com (M.A.K.); hkim@smu.ac.kr (H.-c.K.); 2 Department of Electrical Engineering (ESAT), KU Leuven, 3000 Leuven, Belgium; asaboor@esat.kuleuven.be

**Keywords:** Internet of Things (IoT), Location of Things, target localization, wireless sensor network (WSN), review

## Abstract

The rapid development in wireless technologies is positioning the Internet of Things (IoT) as an essential part of our daily lives. Localization is one of the most attractive applications related to IoT. In the past few years, localization has been gaining attention because of its applicability in safety, health monitoring, environment monitoring, and security. As a result, various localization-based wireless frameworks are being presented to improve such applications’ performances based on specific key performance indicators (KPIs). Therefore, this paper explores the recently proposed localization schemes in IoT. Initially, this paper explains the major KPIs of localization. After that, a thorough comparison of recently proposed localization schemes based on the KPIs is presented. The comparison includes an overview, architecture, network structure, performance parameters, and target KPIs. At the end, possible future directions are presented for the researchers working in this domain.

## 1. Introduction

The Internet is a necessity for billions of people worldwide who need it to complete their daily tasks [1,2]. Furthermore, it provides various entertainment applications such as movies, music, and gaming. One estimate states that more than 58% of the world’s population has access to the Internet to perform such daily tasks. The popularity and growth of the Internet exponentially increased (roughly 1170%) from 2000 to 2020 [3]. It is transforming the world into a global village where people can connect and communicate worldwide using the Internet.

**Figure 1:**
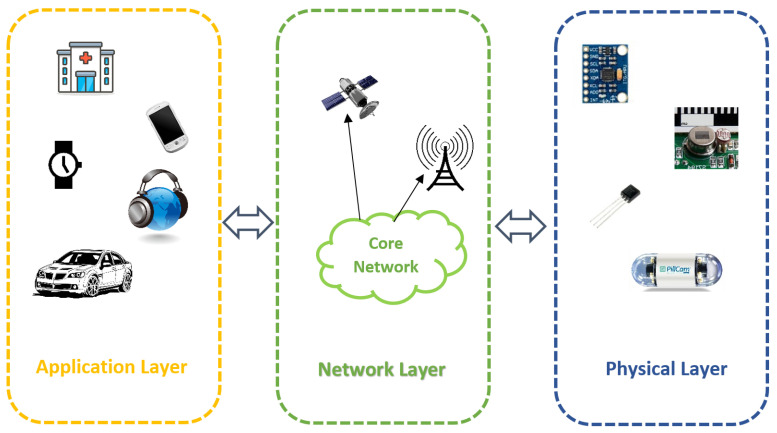
IoT architecture.

The Internet allows different devices and appliances to connect and communicate, which led to a new domain called the Internet of Things (IoT) [4,5,6,7,8]. The IoT architecture consists of three layers: the physical layer, the network layer, and the application layer, as shown in Figure 1. The physical layer consists of various sensors attached to the subject. These sensors collect the data/information from the subject. Generally, the nature of data depends on the IoT application requirements. For example, localization-based applications require monitoring and collection of the locality information of the subject. Likewise, telehealth applications need to monitor the vitals of patients, and agricultural applications measure the temperature. The physical layer forwards the data to the network layer.

The network layer is the middle layer between the application and sensor layer in IoT architecture. The network layer aims to transmit the data/information from the sensors to the application layer. The medium of data transmission (wireless or wired) varies based on the application and requirements. Furthermore, the network layer tries to reduce the network’s data traffic and overheads using optimization techniques. The application layer is the top layer that controls the services provided to the applications. This layer offers an interface to the user to control and manage the IoT devices. Furthermore, it provides services to the application depending on the nature of the application.

IoT is an extension of the Internet that envisions connecting all daily devices to the Internet for communications through interactions or sensing devices. These sensing devices are connected to form a network, termed a wireless sensor network (WSN) [9]. The IoT consisting of WSNs is essential for transforming the world into a smart world [10]. According to the Cisco Annual Internet Report [11], the number of IoT devices will rise from 6.1 billion to 14.7 billion by the end of 2023. Among those 14.7 billion devices, more than 48% of them will assist users in performing daily tasks, such as home automation, security, and tracking applications. Therefore, they act as building blocks in smart cities, smart homes, smart transportation, smart healthcare, smart grids, and smart industry [12,13,14,15,16].

However, there exist numerous challenges in the development of such IoT applications. For example, health-related applications require rapid and reliable data transmission. Monitoring applications in smart environments require energy-efficient and robust protocols [17]. Similarly, there are challenges associated with cost, connectivity, and hardware limitations [18]. However, localization is one key challenges that needs to be addressed in the majority of smart applications. It is the process of acquiring an object or user’s location through intelligent devices (sensors) in an indoor or outdoor environment. It is a critical requirement in most smart applications [19,20]. An exponential increase in smartphones, wristwatches, and other intelligent wireless IoT devices is motivating researchers to develop efficient localization schemes. As a result, we are witnessing a significant rise in localization schemes intended to operate in healthcare, agriculture, environmental work, and habitat monitoring [21,22,23]. Therefore, this paper investigates the IoT-based localization and the proposed schemes [24,25]. The overall contributions of the paper are listed as follows.

1. It provides an overview of localization and its key performance indicators (KPIs).
2. It provides a comprehensive and thorough survey of the recent indoor and outdoor localization schemes. In addition, it highlights the aim and purpose of each localization scheme.
3. It evaluates each scheme with different KPIs such as localization accuracy, energy efficiency, target prediction, target recovery, and security. This evaluation is beneficial for readers aiming to develop a specific localization application.
4. The analysis presents a discussion on IoT localization and highlights the challenges faced by IoT-based localization.
5. It provides open research issues for the researchers working in the localization domain.

A list of acronyms is given in Table 1. The rest of the paper is organized as follows. Section 2 discusses localization and its KPIs. The overall methodology for paper selection is explained in Section 3. Section 4 provides a detailed review of select papers. The discussion is in Section 5. Finally, Section 6 and Section 7 present the future directions and the conclusion, respectively.

## 2. Key Performance Indicators in Localization

Localization is one of the essential applications of smart IoT. Sensor nodes (SNs) keep track of the target and report the location to the user’s application in localization. It requires a single SN or the collaboration of multiple SNs for the tracking of an object. In general, localization is divided into two types: range-based and range-free localization. Range-based localization schemes calculate the distance using multiple geometric and cross grid overlapping techniques such as received signal strength indicator (RSSI), angle of arrival (AoA), and time of arrival (ToA). The anchor nodes’ (AN) positions are known in this technique, and SN determines their locations with reference to AN [26]. In contrast, range-free schemes use hop-count and distance between AN to SN. Furthermore, it uses geometric methods and estimation for localization [27].

**Figure 2:**
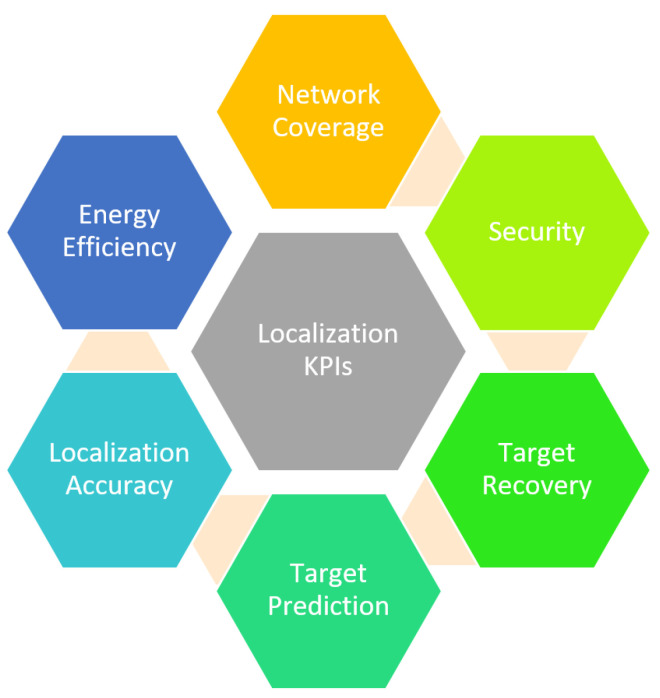
Parameter affecting the performance of localization.

A single SN often causes rapid energy depletion, high computation, and low accuracy, which can be resolved by employing multiple SNs [28]. The IoT unifies multiple transmission techniques, such as Zigbee, Bluetooth, Infrared, WiFi, and the Internet, for efficient target localization and tracking. The choice of SNs may vary concerning applications such as radio frequency identification (RFID) in indoor tracking and acoustic sensors array for underwater localization. This inter-linkage and smooth cooperation among IoT devices is the cornerstone for its efficient and reliable working. Hence, the IoT-based network is very heterogeneous in SNs and communication protocols [29,30]. It gives rise to several challenges due to non-standardization or unifying of different protocols. However, depending on the target application, these SNs have several KPIs: energy consumption, rapid power depletion, computation, and security. The scope of this review is to highlight and evaluate only the localization KPIs in IoT devices. Therefore, we have not considered KPIs related to other IoT applications. The critical KPIs of target localization are illustrated in Figure 2.

### 2.1. Network Coverage

Coverage is associated with the sensing range of the WSN. In localization, it is the extent of a target in a network under the surveillance of a SN. Therefore, it ensures the uniformity of the SNs and avoids black holes in the overall network.

### 2.2. Security

Generally, the WSN is deployed in an external environment such as a forest, a building, or a battlefield. It transmits information over the wireless channel vulnerable to various security attacks such as jamming, spoofing, misdirection, and Sybil attacks. Therefore, security ensures information reliability, authentication, and integrity, and avoids security attacks such as flooding and spoofing.

### 2.3. Target Recovery

Sometimes, a target gets lost due to a prediction error, a communication error, a black hole, or an SN failure. It leads a target to pass through a specific region undetected and compromise overall tracking accuracy. Target recovery tries to retrieve such target(s) in minimum time using an optimal number of sensors to ensure overall energy efficiency.

### 2.4. Target Prediction

The prediction in localization aims to predict the location of the mobile target. It results in improving the efficiency of target localization. In general, this parameter measures the overall probabilities of true-positive and false-negative results.

### 2.5. Localization Accuracy

Localization accuracy specifies the accuracy by which the position of the target is determined. In localization, the location of the SN is critical, as a minor location error generates worthless data.

### 2.6. Energy Efficiency

SNs run on batteries that are non-rechargeable and sometimes located in a non-changeable environment [31]. Some SNs consume more energy during the localization due to idle listening, overhearing, and packet collision. The energy efficiency and lifetime of SNs pose severe issues in WSN, specifically in sensitive target tracking applications.

This paper aims to analyze the recent localization schemes based on the KPIs mentioned above. A detailed methodology of paper selection is presented in the next section.

## 3. Methodology

**Figure 3:**
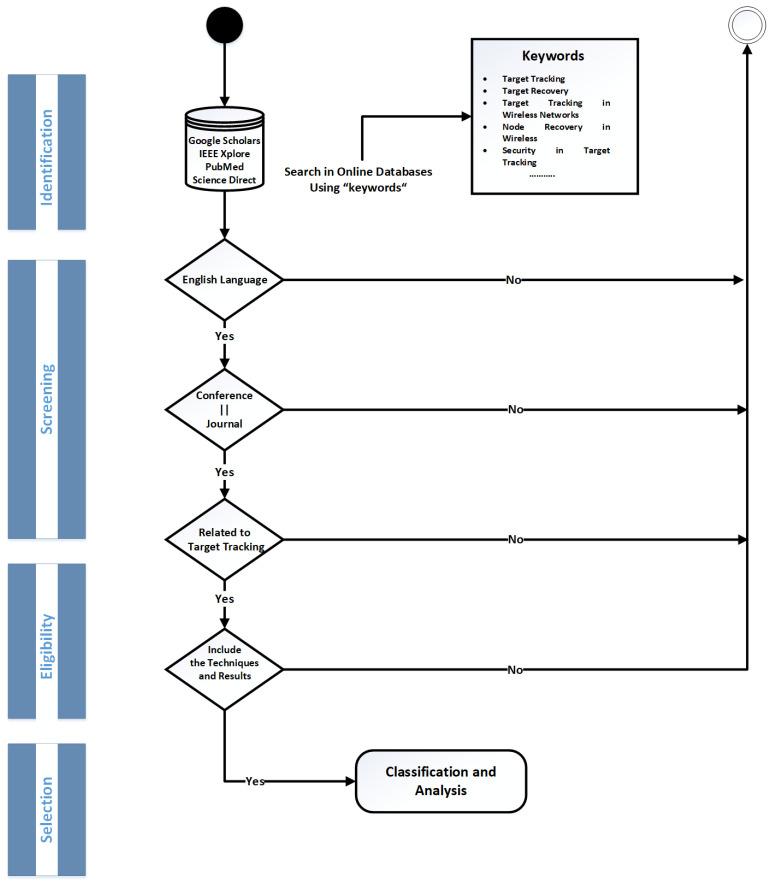
Proposed methodology.

The Preferred Reporting Items for Systematic Reviews and Meta-Analyses (PRISMA) approach is used in this review [32]. An overview of the proposed methodology is presented in Figure 3. The overall methodology consists of four phases:

- Identification.
- Screening.
- Eligibility.
- Selection.

The identification process consists of the initial selection of the papers based on the abstracts. Different science libraries, such as Google Scholar, IEEE Xplore, Pub Med, and Science Direct, were used using various strings. Table 2 provides the list of strings for searching desired papers. Based on the above-mentioned strings, more than 1000 documents were obtained from multiple academic databases. Only documents newer than 2010 were considered in the identification process. The obtained documents were filtered out based on the title in the initial screening process. A total of 249 papers were selected after applying various screening filters, such as the English language. The 21 duplicates were also removed, which resulted in 228 documents for the eligibility check criteria. All the papers in this phase were read carefully, and the following parameters made them eligible for the selection process:

- The paper should be published in journal or conference.
- The papers should consider the target tracking.
- The papers should present a concrete methodology and results.

Finally, a total of 40 papers were selected after eliminating the papers based on the above-mentioned criteria. These papers were used as a part of the review and further analyzed. The following section provides a complete analysis of selected publications using this methodology.

## 4. Review of Location Aware Schemes in IoT

A comparison of the state-of-the-art studies is given in Table 3. From the table, it is clear that most of the existing surveys target a particular localization domain, i.e., outdoor or indoor.

The main focuses of already published surveys were accuracy, energy efficiency, target prediction, and security. They lack some critical KPIs, such as recovery, prediction, security, and localization with smart gadgets, i.e., smartphones. Only 28% of publications covered prediction; 35% covered security and localization with smartphones. Simultaneously, no published survey covered the target recovery KPI, which is an important indicator that affects the overall performance of the IoT-based localization scheme.

Furthermore, these surveys lacked detail and generic discussion in terms of protocols and techniques for localization schemes. Therefore, there is a need for a cross-domain survey that puts forward an in-depth discussion on the IoT-based localization scheme. As mentioned in Section 3, a total of 40 papers were selected using the PRISMA approach. The key ideas of the selected papers are presented in Table 4. In addition to that, the selected studies were analyzed based on publishing details and target applications.

### 4.1. Distribution Based on Publishing Year

**Figure 4:**
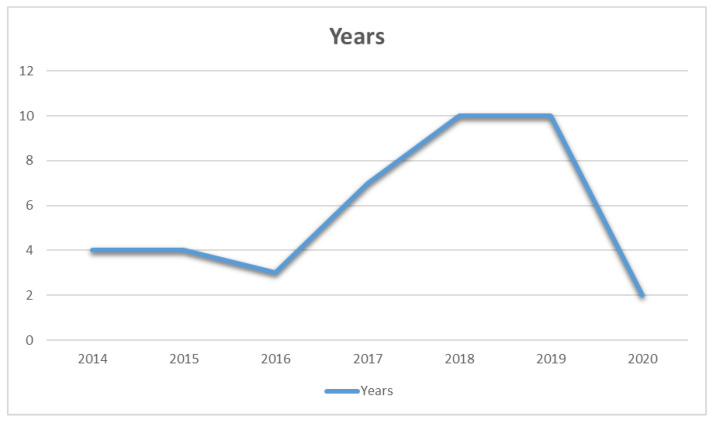
Distribution based on publication year.

We aim to highlight the recent trends in target localization. Therefore, the papers from the last seven years (2014 and onward) are considered. The yearly distribution of the selected publications is presented in Figure 4. From the figure, it is depicted that there was less interest in localization in earlier years. However, it started rising from 2017. The last three years of research (2017–2019) comprised 67% of publications selected for this review. 2020 was just beginning when the papers were shortlisted. However, based on the trend, more contributions are expected in this domain than in past years.

### 4.2. Distribution Based on Publication Venue

**Figure 5:**
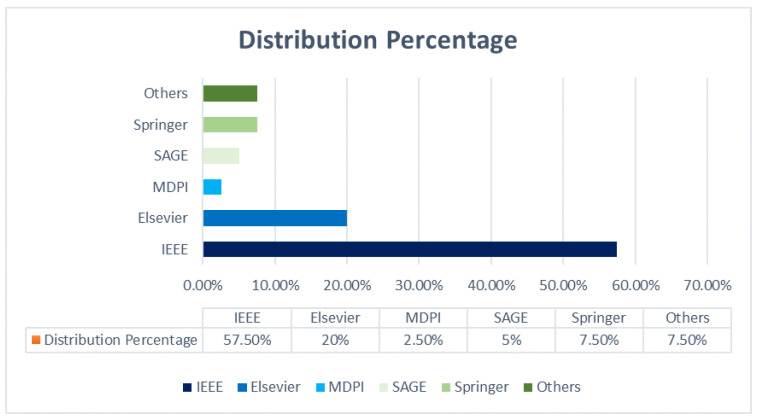
Distribution based on publishing venue.

This section aims to highlight the publication venue distribution. Our study includes various publication venues, such as IEEE, Elsevier, MDPI, and SAGE. The distribution of publications concerning the venues is presented in Figure 5. It was found that IEEE and Elsevier support most publications in the domain of target tracking—57.5% and 20%, respectively. Therefore, these two venues are recommended for localization in IoT.

### 4.3. Distribution Based on Publication Type

**Figure 6:**
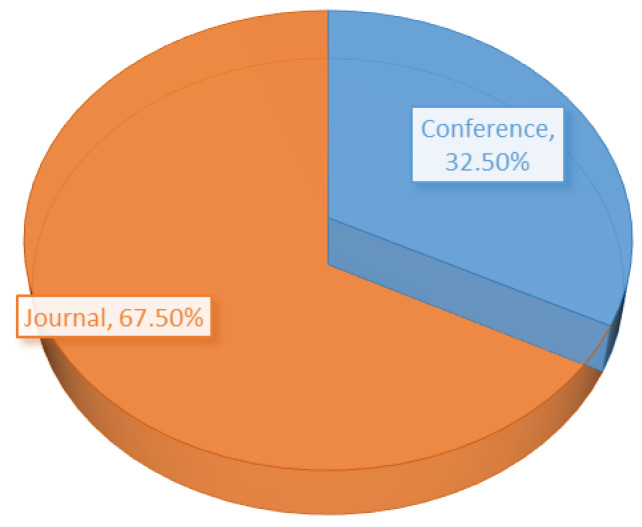
Distribution based on publication type.

As mentioned in Section 3, we have only considered conference proceedings and journal articles for the review. The distribution percentages of those publication types are presented in the form of a pie chart. From the Figure 6, it is clear that our results are mainly backed up by journal articles (67%).

### 4.4. Analysis Based on Localization KPIs

There are various localization KPIs, such as energy efficiency, localization accuracy, target prediction, target recovery, and security. Every paper is trying to address single or multiple KPIs. Table 5 lists the selected publications along with the target KPIs. The lifetime of sensors is a major concern in WSNs, as battery replacement is a tiring and time-consuming job. Additionally, low-battery or abandoned sensors can halt the performance of the overall network in emergencies.

**Figure 7:**
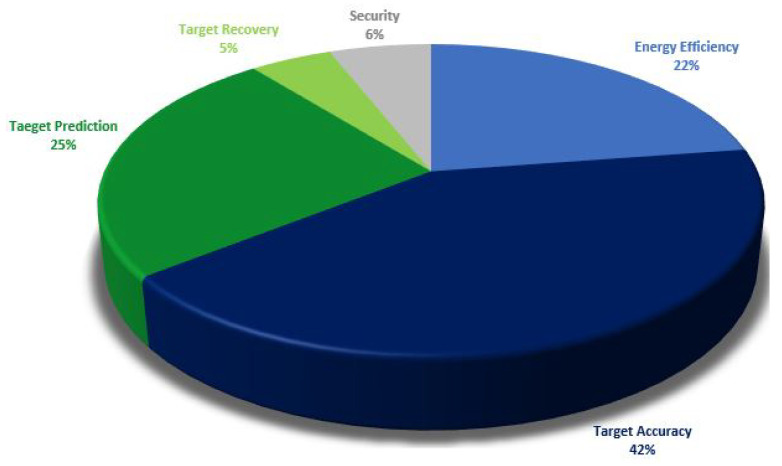
Distribution based on target challenges.

Furthermore, accurate predictions and identification of targets are desired in location-aware schemes. Therefore, energy efficiency, tracking accuracy, and target prediction are the most researched KPIs in target tracking of WSNs, as shown in Figure 7. In contrast, target recovery and security were explored in only 11% of the papers selected. These are also important aspects of localization that need the researcher’s attention in the future. In addition to the above analysis, a general overview of all the papers, including the proposed approach, network structure, number of targets, and performance parameters, is presented in Table 6.

## 5. Discussion

The above section provides a review of the papers selected. In addition to that, we have learned several lessons during our analysis. For example, a lot of research has been done in pursuance of making WSNs smart and energy-efficient. A system that consumes much energy is ill-suited for most applications. Therefore, energy consumption is one of the core issues in terms of smart environment and localization. It is directly linked to the latency and performance of localization. Some influential factors are the number of nodes and transmission range. The number of SNs involved in the target localization will significantly affect localization accuracy and energy consumption. The higher the involvement of SNs in localization, the higher the localization accuracy and energy consumption. Hence, we must maintain a balance between accuracy and overall energy consumption. The transmission range is also a leading cause of energy consumption. Higher signal power results in improving the signal range of the SN. However, it also results in quick energy depletion. Thus, a trade-off between signal range and energy consumption is required. Some other factors which affect energy consumption are interference and periodic beacon transmission.

Accuracy is fundamental in terms of localization and its applications. In recent years, extensive research has been done to improve localization accuracy. However, most of the schemes are environment-specific (indoor or outdoor), which affects their widespread applicability. In an indoor environment, the efficiency and accuracy of localization show notable reductions in the presence of objects, noise, and the multi-path effect. Hence, we need such schemes to limit the effect of surrounding objects and the multi-path impact on accuracy. Contrarily, the localization accuracy is primarily dependent on GPS and radio frequency (RF) in outdoor scenarios. However, high accuracy is not achievable, and batteries are depleted rapidly. The researchers are mainly focusing on indoor localization. Thus, outdoor localization is still an open issue. There is a need to design a scheme that is independent of the environment in terms of performance.

Different prediction algorithms are used to improve target localization. However, these power-hungry algorithms reduce the lifetimes of SNs. In recent years, researchers have presented different low duty cycle prediction schemes in which the SNs close to the target remain functional, while others are kept in a sleep state [49,59]. However, the prediction algorithm must be accurate because failure to accurately predict the next target location might drastically affect the localization accuracy and performance. The error in the prediction algorithm due to varying speed results in target loss. To recover the lost target, all SNs should go in an active state to track and recover the lost target. However, that results in the rapid depletion of energy. Hence, multiple energy-efficient recovery schemes have been presented using different filters such as KF, UKF [54,64]. Furthermore, some researchers have used the genetic algorithm for target tracking and recovery [83].

Security is also a major concern in mission-critical applications such as battlefield monitoring, soldier monitoring, and telehealth monitoring. Often these SNs are deployed in a hostile environment that can be easily corrupt and exploited by intruders. To overcome this, some authors proposed k-mean clustering for node authenticity in [76], in which only trustworthy SNs are used for localization and other operations. Some studies suggest the use of cryptography or digital signature base security [62,84]. Network coverage is one of the issues in WSN and directly related to localization performance. Non-uniform distribution of SNs would result in holes in the region of SN deployment. Multiple network techniques have been presented since. We differentiate network structures into two types: tree structure; cluster structure. In a tree-based network structure, deployed SNs form a logical tree architecture where data travel from leaf SN to root SN. This process preserves energy by avoiding packet flooding and broadcasting.

However, all the nodes in a cluster structure are combined to form a cluster with one or multiple cluster heads (CHs). Cluster-based topology improves the scalability and bandwidth efficiency as compared to the other topologies. A CH reduces the packets transmitted to a base station, improving energy consumption, bandwidth usage, and security. Clustering can be either static or dynamic. Static clusters are created at network establishment time and remain fixed during the whole lifetime of the network. Apart from its simplicity, it has several drawbacks, such as the life of the whole cluster being dependent upon the CH. Additionally, it eliminates the possibility of data sharing and collaboration among clusters. In contrast, dynamic clusters form on runtime as the target travels. They are more flexible and energy-efficient than static clustering, because the clusters are formed when the necessity arises. However, they face data redundancy and interference issues.

## 6. Future Directions

Localization and its applications have gained much attention from researchers in the past few years. Different schemes have been presented to improve energy efficiency, localization accuracy, target prediction, and security. Despite that, many aspects still need improvements to enhance the capabilities of these applications. In this section, we discuss the open research challenges and possible future directions in this domain.

- *Environment Independence*—The majority of studies focus on either indoor [87,88,89,90] or outdoor localization [91,92,93]. This environment-centric application’s nature limits the applicability and widespread use in real-life scenarios. Hence, there is a dire need for environment-independent localization algorithms that are feasible for both indoor and outdoor applications [94,95,96]. This will eventually improve the adaptability of these applications in practical applications such as emergency evacuations, shipment/cargo tracking, and mission-critical applications.
- *Security and Privacy*—Security is one of the least explored challenges in IoT-based localization applications. However, this domain requires attention, as a user reveals far more personal information (via wireless channels) when using such applications. Compromising user location can be dangerous and life-threatening for some IoT services and applications, such as health, industry, and defense. For example, in an industrial environment, compromises in security and privacy might lead to a violation of confidential information related to the company’s product. Henceforth, the rapid increase in cybersecurity challenges and lack of standardization for basic privacy mechanisms make it an open research problem [12,97]. Multiple authors have suggested the embedding of deep learning techniques to improve the security in IoT-based Localization applications [98,99,100]. Additionally, encryption algorithms and digital signatures using public and private keys can improve the resilience against external attacks [101,102]. However, the extent of security by using minimal resources is still a big challenge.
- *Energy Efficiency*—Generally, the SNs are tiny with irreplaceable batteries that make them resource-scarce. Due to this, energy consumption is one of the primary challenges in localization applications. For that, some energy-efficient techniques [103,104,105] have been presented. These SNs are mostly deployed in external environments, which motivates the use of energy harvesting [106,107]. In this context, a few energy harvesting techniques for the prolonging of network lifetime have been presented in the literature [108,109,110,111]. Furthermore, machine learning (ML) algorithms integrated with energy harvesting technology are also candidates for improving network lifetime and performance by predicting the amount of energy to be harvested from ambient in a specific duty cycle [112,113].
- *Accuracy*—Extensive research has been done on localization accuracy in IoT. However, most of the proposed schemes neglect the resource-scarce nature of the SN, thereby making them ineligible for real-life applications. For example, GPS and cellular data improve accuracy but deplete the battery rapidly. Additionally, the accuracy is compromised by the shadowing effect. Therefore, the implementations of error-resilient and vigorous mechanisms such as adaptive scheduling algorithms, prediction, and localization optimization schemes can be developed to improve localization accuracy [114]. Cloud computing with better prediction algorithms (process at cloud) can lead to accurate localization schemes while consuming minimal resources.
  In the literature, some authors proposed ML-based localization schemes [115,116,117] to improve the localization accuracy. By integrating ML with the localization, the progressive likelihood surpassed the posterior likelihood. In addition to that, this could also assist with predicting the target’s next possible location. Henceforth, it will activate only those SNs which are closest to that prediction.
- *Data Flow*—The data flow varies from scenario to scenario, i.e., normal or emergency. During a natural catastrophe/disaster, communication is an essential part of an emergency evacuation. Likewise, IoT devices are commonly used in healthcare—e.g., remote monitoring [118,119] and body posture monitoring [120,121]. The interruption in data flow can risk the patient’s life, which makes IoT inadequate for the health sector. Therefore, such schemes need to be developed which can perform better in emergency scenarios [42,122]. Priority-driven approaches have the potential to overcome such problems. For example, the IEEE 802.15.6 WBAN standard and its compatible devices can resolve this problem. This standard consists of eight priority levels, and the data flow of emergency traffic is always prioritized [123].
- *Data Association*—Tracking multiple targets is a challenging task because of the differences in speed and direction of targets. Due to the presence of multiple targets, SNs receive multiple pieces of target information [124,125,126]. The main hurdle is to differentiate which information belongs to which target. This improbability in information results in the data association problem. Therefore, distinguishing the data of specific targets in the presence of multiple targets requires attention. Various classification algorithms, such as support vector machines (SVM), decision trees (DT), and neural networks offer the potential to resolve this problem.

## 7. Conclusions

IoT’s intrinsic nature makes it deployable everywhere—e.g., roads, homes, forests, and even underwater. Localization is one of the widely used applications of IoT. It is used in diverse fields, such as healthcare, security surveillance, monitoring, and vehicle tracking. Localization offers application-oriented KPIs such as energy, target prediction, network coverage, and security. Therefore, numerous studies/schemes have been presented in the literature to address one or multiple KPIs. This paper presented a detailed review of the recently proposed localization schemes. The review examined different localization schemes, key ideas, propositions, network architectures, performance parameters, and target KPIs. It is noted that the dynamic CH selection improves the flexibility and energy efficiency of the scheme.

Furthermore, the paper showed that there is always a trade-off among various KPIs, i.e., target recovery and energy efficiency. The review also highlighted that most of the selected schemes improved the accuracy and energy efficiency of the localization applications. In contrast, security and target recovery were less explored. Lastly, we highlighted the open research challenges to improving the performance of localization in IoT.

## Figures and Tables

**Table 1:** List of important acronyms.

Acronym	Extended Meaning	Acronym	Extended Meaning
WSN	Wireless Sensor Network	SN	Sensor Node
AN	Anchor Nodes	IoT	Internet of Things
NH	Neighbourhood Heuristics	ETX-NH	Expected Transmissions with Neighbourhood Heuristics
PF-DLSTA	Particle Filtering based Dynamic Lookahead Tree Based Tracking Algorithm	WSHAN	Wireless Sensor Hole Aware Network
BCTT	Boundary Static Clustering Target Tracking	SCDCH	Static Cluster and Dynamic Cluster Head
KPI	Key Performance Indicator	ML	Machine Learning
PPHD-MMA	Particle filter based Probability Hypothesis Density incorporated with Multipath-to-Measurement Association	VGTR	Virtual Grid-based Target Recovery
FSM	Fuzzy Sensing Model	DCTC	Dynamic Convoy Tree-based Collaboration
RFID	Radio frequency identification	GPS	Global Positioning System
JPDA	Joint Probablistic Data Association	PUESRF	Particle wise Update version of Ensemble Square Root Filter
IPAH	Improved Prediction based Adaptive-Head	DCTT	Distributed Cluster-based algorithm for Target Tracking
PCTT	Prediction-based Clustering algorithm for Target Tracking	PSO	Particle Swarm Optimization
ASMT	Augmented State-based Multi-target Tracking	GTPM	Game Theory Payoff Matrix
SRPTT	Secure and Reliable Prediction-based Target Tracking Protocol	AEKF	Adaptive Extended Kalman Filter
DHSCA	Dual Head Static Clustering Algorithm	ACDF	Adaptive Consensus based with the Distributed estimator Filter
UKF	Unscented Kalman Filter	4WS4WD	Four-wheel-steering and four-wheel-driving
HNTA	Hybrid Network Tracking Algorithm	POMDP	Partially Observed Markov Decision Process
EEA-IAH	Energy-Aware Improved Adaptive-Head	LPPT	Low Prediction Precision requirement Target tracking
CLTA	Cooperative Localization and Tracking Algorithm	FTS	Fuzzy Tree System
DMGIF	Distributed Multiple Gaussian Information Filter	ARIMA	Auto Regressive with Moving Average
ELM	Extreme Learning Machine	LEMon	Localization Employing a location-unaware MObile unit
RSSI	Received Signal Strength Indication	LEMon-M	LEMOn for localization Matching
TS PM-PHD	Two Steps Product Multi-sensor Probability Hypothesis Density	MC-SSN	Mission Critical Sensors and Sensor Networks
AIE-MCMCDA	Augmented Input Estimation Markov Chain Monte-Carlo Data Association	CS-BnB	Convex hull Sampling based Branch and Boun
AMCL	Adaptive Monte Carlo Localization	IMM	Interactive Multi Model

**Table 2:** Strings used in search engines.

Academic Library	Search String
Google Scholar	⇒ Target Tracking ⇒ Target Localization in wireless Network ⇒ Routing protocol for target tracking ⇒ Target recovery in wireless networks ⇒ Target Localization and artificial intelligence ⇒ Single Target Tracking ⇒ Multi Target Tracking ⇒ Target Tracking Using Machine Learning ⇒ Prediction Based target Tracking ⇒ Energy Efficient Tracking ⇒ Target Tracking Applications
IEEE Xplore	⇒ (((“All Metadata”:target) AND “All Metadata”:tracking) OR “All Metadata”:wireless networks) //Filters Applied: 2010–2020 ⇒ (((“All Metadata”:target) AND “All Metadata”:tracking) OR “All Metadata”:target localization) //Filters Applied: 2010–2020 ⇒ (((“All Metadata”:target tracking) OR “All Metadata”:single target) OR “All Metadata”:multi target) //Filters Applied: 2010–2020 ⇒ (((“All Metadata”:target tracking) AND “All Metadata”:efficient) OR “All Metadata”: prediction) //Filters Applied: 2010–2020
PubMed	⇒ target[Title/Abstract] AND tracking[Title/Abstract] OR wireless networks[Title/Abstract] AND ("2010/01/01"[PDAT]: “2020/01/01”[PDAT]) ⇒ target[Title/Abstract] AND tracking[Title/Abstract] OR efficient [Title/Abstract] AND (“2010/01/01”[PDAT]: “2020/01/01”[PDAT]) ⇒ target[Title/Abstract] AND tracking[Title/Abstract] OR machine learning [Title/Abstract] AND (“2010/01/01”[PDAT]: “2020/01/01”[PDAT])
Science Direct	⇒Target Tracking’ and Wireless Networks or prediction. Limited to research articles, conference abstracts. ⇒Target Tracking’ and Wireless Networks or efficient. Limited to research articles, conference abstracts. ⇒Target Tracking’ and Wireless Networks or applications. Limited to research articles, conference abstracts.

**Table 3:** Comparison between this paper and published surveys.

References	Indoor Localization	Outdoor Localization	Smartphone Localization	Security	Energy Efficiency	Accuracy	Target Recovery	Target Prediction
[33]	✓				✓	✓		
[34]	✓				✓	✓		
[35]	✓				✓	✓		
[36]	✓			✓	✓	✓		✓
[37]	✓					✓		
[38]	✓			✓		✓		
[39]		✓			✓	✓		
[40]	✓	✓			✓	✓		✓
[41]	✓	✓				✓		
[42]	✓	✓		✓		✓		
[43]	✓		✓	✓	✓	✓		
[44]	✓		✓			✓		
[45]	✓	✓	✓			✓		✓
[46]	✓		✓		✓	✓		✓
Ours	✓	✓	✓	✓	✓	✓	✓	✓

**Table 4:** Key ideas of selected papers.

Name	Overview
Delaney et al. [47]	This paper presents an energy efficient routing protocol using NHs model for tree structured WSN. Apart from energy efficiency, the proposed solution has the ability to present good results in a lossy network environment.
Alaybeyoglu et al. [48]	This paper presents an efficient tracking scheme for high speed targets. Additionally, the proposed scheme helps in reducing the target miss ratio during the whole tracking lifecycle.
Mirsadeghi et al. [49]	This paper presents an energy efficient prediction based target tracking scheme for WSN. The node closest to the object or with the highest energy is selected as a CH to prolong the network lifetime.
Patil et al. [50]	This paper presents an energy efficient WSHAN to improve the efficiency of target tracking target recovery.
Rouhani et al. [51]	This paper presents a solution to resolve the boundary target tracking issues using static clustering. The proposed solution is energy efficient, reasonably accurate and reliable in terms of target tracking.
Wahdan et al. [52]	This paper presents a hybrid solution of static networking clustering and dynamic CH. The dynamic CH uniformly utilize the energy of member SNs to prolong the network lifetime and prediction.
Zhou et al. [53]	This paper presents a fusion of MMA and PPHD for multi-target tracking in an urban area. Additionally, K-mean clustering is used to calculate the number of targets at any given time. The proposed scheme results in the tracking of dynamically changing unknown numbers of targets in urban areas.
Amudha et al. [54]	This paper presents a multi camera based scheme for target tracking. In this scheme, the camera near the mobile target is activated while all other cameras remain in a sleep state to conserve energy. In contrast, all the cameras are activated when a target is lost to improve the tracking.
Bhowmik et al. [55]	This paper presents an algorithm is to improve the overall coverage and target tracking. In addition to that, the proposed algorithm uses the FSM based RSSI tracking algorithm to make it more energy efficient.
Jinan et al. [56]	This paper presents a multi-model framework based on the PUESRF and JPDA. It results in improving the accuracy and precision of data that makes target tracking consistent.
Darabkh et al. [57]	This paper presents an adaptive CH algorithm with an aim to achieve a better target tracking by efficiently electing CH and cluster members. The proposed algorithm is energy efficiency and improves the network scalability.
Khakpour et al. [58]	This paper presents a fusion of DCTT and PCTT against vehicular tracking in a Vehicular Ad-hoc Network. To improve the target prediction, The DCTT performs in a distributed manner while PCTT is used for a centralized prediction algorithm.
Joshi et al. [59]	This paper presents a static cluster based target tracking for the prediction that is independent of wireless network architecture (homogenous or heterogeneous). The proposed scheme uses a linear prediction technique to calculate direction and speed to improve the target prediction.
Xiao et al. [60]	This paper presents a novel ASMT using Bayesian estimation to efficiently track multiple targets. The ASMT provides high accuracy, precision based multi-target tracking, less computation and solves the data association problem in WSN very efficiently by using location state and velocity state.
Silva et al. [61]	This paper presents an energy efficient scheme with the ability to detect and highlight the fake node positioning and bogus data flooding.
Oracevic et al. [62]	This paper presents a SRPTT algorithm to prevent the rouge SN from faking its location or flooding the bogus packets in a WSN. The SRPTT maintains a balance between security and mobile target tracking by employing a reputation concept.
Alshamaa et al. [63]	This paper presents a novel zoning based localization technique for indoor target tracking. The proposed technique develops a belief function by combining fingerprint based target observation and evidence associated with sensor mobility to improve the accuracy of target tracking.
Chen et al. [64]	This paper presents an adaptive extended kalman filter to remove and update the noise covariance. The proposed solution results in improving the accuracy and reliability of target tracking.
Panag et al. [65]	This paper presents a DHSCA to uniformly utilize sensors during the tracking. The proposed algorithm simplifies the set-up phase time of the network resulting in reducing the overhead of the network.
Zhang et al. [66]	This paper presents a dynamic clustering-based adaptive filtering scheme for target tracking in a WSN. The proposed scheme consists of two stages hierarchal data aggregation technique, which results in accurate and energy efficient target tracking.
Qian et al. [67]	This paper presents an AUKF algorithm to enhance the robustness and accuracy of the recovery mechanism. The AUKF fine-tunes the noise covariance matrix to increase the accuracy and robustness of the recovery mechanism. The vigorous scheduling of static and mobile SNs improves the tracking probability with less energy consumption.
Zhang et al. [68]	This paper presents an algorithm based on a hybrid sensor network to estimate the target region via static sensors. Additionally, a movement algorithm is presented for nodes to select the location. The proposed solution results in conserving the energy by reducing the target tracking sensors.
Li et al. [69]	This paper presents a sensor selection technique based on POMDP to reduce the sensor selection lagging. It results in improving the target tracking accuracy and reliability.
Darabkh et al. [70]	This paper presents an error and Energy-aware cluster head selection algorithm to improve the target localization. The proposed algorithm improves energy consumption and simplifies the selection of cluster members. Additionally, it reduces the packets overhead by minimizing the transmission of control messages.
Liu et al. [71]	This paper presents energy efficient scheme with low prediction accuracy. Apart from energy efficiency, it reduces the target miss rate probability.
Luo et al. [72]	This paper presents a scheme to improve the target tracking for an indoor environment using a CLTA.
Yu et al. [73]	This paper presents a mobile node-based target tracking scheme to enhance the target tracking accuracy and transmission reliability.
Vallas et al. [74]	This paper presents a Gaussian filter-based multi-sigma point filter to reduce the curse of dimensionality in high dimension systems. Furthermore, it improves the efficiency of tracking the multiple targets in a WSN.
Ghodousi et al. [75]	This paper presents an energy efficient tracking scheme using ARIMA and UKF. The ARIMA, after observing target in equal interval, predicts its future location while UKF estimates the target location. The proposed scheme preserves the energy of SNs and improves the network lifetime.
Liang et al. [76]	This paper presents a trust-based distributed KF scheme for secure and reliable target tracking.
Khan et al. [77]	This paper presents a dynamic clustering-based verifiable multi iteration scheme to improve target tracking. The proposed scheme improves the accuracy and reliability of tracking.
Liu et al. [78]	This paper presents an object localization scheme to provide better localization results on the sequences undergoing shape deformation and illumination changes.
Nguyen et al. [79]	This paper presents a solution to improve the accuracy of target tracking in harsh radio environments. The proposed scheme is efficient in both indoor and outdoor environments.
Ullah et al. [80]	This paper presents an underwater target tracking scheme intending to achieve energy efficiency and tracking accuracy.
Alberto et al. [81]	This paper presents a multi-model tracking system by unifying fingerprint-based tracking with neural networks. The proposed system also employs a Gaussian outliers filter with neural networks to further improve the tracking accuracy.
Liu et al. [82]	This paper presents a scheme for tracking multiple targets in a harsh environment accurately and precisely.
Liu et al. [83]	This paper presents an AFS for accurate and efficient target tracking. The proposed scheme is robust and fault-tolerant with a low target loss rate. Moreover, PSO is used to fine-tune and improve the overall tracking performance.
Mahmoudreza et al. [84]	This paper presents a solution to tackle the multiple target tracking problems with accurate data association. It results in the prevention of false alarms.
Li et al. [85]	This paper presents a hybrid solution to provide accurate and reliable localization in harsh manufacturing workshops.
Reisinger et al. [86]	This paper presents an IMM tracking scheme unified with UKF to track the targets efficiently.

**Table 5:** KPIs Addressed in Selected Papers.

Name	Energy Efficiency	Localization Accuracy	Target Predication	Target Recovery	Security
Delaney et al. [47]	✓				
Alaybeyoglu et al. [48]	✓	✓	✓		
Mirsadeghi et al. [49]	✓	✓	✓		
Patil et al. [50]	✓	✓	✓	✓	
Rouhani et al. [51]	✓	✓			
Wahdan et al. [52]	✓	✓	✓		
Zhouet al. [53]		✓	✓		
Amudha et al. [54]	✓	✓		✓	
Bhowmik et al. [55]	✓	✓			
Jinan et al. [56]		✓	✓		
Darabkh et al. [57]	✓	✓	✓		
Khakpour et al. [58]		✓	✓		
Joshi et al. [59]		✓	✓		
Xiao et al. [60]		✓			
Silva et al. [61]	✓		✓		✓
Oracevic et al. [62]		✓	✓		✓
Alshamaa et al. [63]		✓			
Chen et al. [64]		✓	✓		
Panag et al. [65]	✓				
Zhang et al. [66]	✓	✓			
Qian et al. [67]			✓	✓	
Zhang et al. [68]	✓	✓			
Li et al. [69]	✓		✓		
Darabkh et al. [70]	✓	✓			
Liu et al. [71]	✓	✓	✓		
Luo et al. [72]		✓			
Yu et al. [73]		✓	✓		
Vallas et al. [74]		✓	✓		
Ghodousi et al. [75]	✓	✓	✓		
Liang et al. [76]		✓			✓
Khan et al. [77]	✓	✓			✓
Liu et al. [78]		✓	✓		
Nguyen et al. [79]		✓			
Ullah et al. [80]	✓	✓			
Alberto et al. [81]		✓			
Liu et al. [82]		✓			
Liu et al. [83]		✓	✓	✓	
Mahmoudreza et al. [84]		✓			✓
Li et al. [85]		✓			
Reisinger et al. [86]		✓	✓		

**Table 6:** Overview of selected papers.

Ref.	Proposed Approach	Network Structure	Number of Targets	Performance Parameters	Tool
[47]	ETX-NH	Tree	Single	PDR: 96%	TOSSIM
[48]	PF-DLSTA	Tree	Single	N/A	NS2
[49]	Low Power Target Prediction Mechanism	Dynamic Cluster	Single	MR: 0.69%	N/A
[50]	WSHAN	Dynamic Cluster	Single	EE: 37%	MATLAB
[51]	BCTT	Static Cluster	Single	EE: 48%	Omnet++
[52]	SCDCH	Static Cluster	Single	N/A	MATLAB
[53]	PPHD-MMA	Dynamic Cluster	Multiple	N/A	N/A
[54]	VGTR	Dynamic Cluster	Single	TMR: 99% reduction	MATLAB
[55]	DCTC with Fuzzy Sensing	Tree	Single	N/A	TinyOS and nesC
[56]	JPDA, PUESRF	Dynamic Cluster	Multiple	N/A	N/A
[57]	IPAH	Dynamic Cluster	Single	EE: 40% improved, LE: 52% improved	MATLAB
[58]	DCTT, PCTT	Static Cluster	Single	N/A	NS2 + TOSSIM
[59]	Prediction based object tracking algorithm	Static Cluster	Single	PA: 99%	NS2
[60]	ASMT	Static Cluster	Multiple	FR: >14%	N/A
[61]	GTPM	Dynamic Cluster	Single	N/A	NS2
[62]	SRPTT	Static Cluster	Single	N/A	Java Simulator
[63]	Extended observation model, 2^nd^ mobility model	Static Cluster	Single	N/A	N/A
[64]	AEKF	Static Cluster	Single	RMSE: 32.53%	N/A
[65]	DHSCA	Static Cluster	Single	N/A	Fortran PowerStation 4.0
[66]	ACDF	Dynamic Cluster	Single	N/A	N/A
[67]	AUKF	Static Cluster	Single	N/A	MATLAB
[68]	HNTA	Hybrid Cluster	Multiple	N/A	N/A
[69]	Adaptive sensor selection algorithm with POMDP	Dynamic Cluster	Multiple	N/A	N/A
[70]	EEA-IAH	Dynamic Cluster	Single	N/A	MATLAB
[71]	LPPT	Static Cluster	Single	Reduce MR: 36.34%, EE: 5.2 times	Omnet++
[72]	CLTA	Dynamic Cluster	Single	LE: 0.65 m	MATLAB
[73]	FTS	Tree	Single	LE: >50 improvement	MATLAB
[74]	DMGIF	Dynamic Cluster	Multiple	N/A	N/A
[75]	ARIMA, AUKF	Dynamic Cluster	Single	N/A	Opnet + MATLAB
[76]	Trust-based distributed Kalman filtering.	Dynamic Cluster	Single	N/A	N/A
[77]	Dynamic cooperative multilateral sensing	Dynamic Cluster	Single	LE: 19% improved	MATLAB
[78]	ELM compressive sensing	Dynamic Cluster	Single	N/A	MATLAB
[79]	LEMon, LEMon-M	Static Cluster	Single	Outdoor and Indoor LE: 10 m and 2 m improved	N/A
[80]	Distance and angle-based localization	Dynamic Cluster	Single	LE: 90% improved, ABL: 104.9 m	N/A
[81]	SWiBluX	Dynamic Cluster	Single	LE: 45% improved	N/A
[82]	TS PM-PHD	Dynamic Cluster	Multiple	N/A	N/A
[83]	AFS for MC-SSN	Tree	Single	LE: <0.2%	N/A
[84]	AIE-MCMCDA	Dynamic Cluster	Multiple	LE: 0.39–4.12%	N/A
[85]	CS-BnB, BnB-AMCL	Dynamic Cluster	Single	LE: 0.005 m/0.111 deg	4WS4WDr
[86]	IMM, UKF	Dynamic	Multiple	EE: 4 times	N/A

## Data Availability

Not applicable.
